# How do intensive care physicians wish to die? A cross-sectional study on end-of-life preferences and the role of palliative care

**DOI:** 10.3389/fmed.2026.1872490

**Published:** 2026-07-06

**Authors:** Yaşar Gökhan Gül, Sinan Uzman, Ali Haspolat, Nurşen Tanrıkulu, Ersi Abacı Kalfoğlu, Ali Şefik Köprülü

**Affiliations:** 1 Department of Anesthesiology and Reanimation, Istanbul Medipol University Medicine Faculty Medipol Mega Hospital, Istanbul, Türkiye; 2 Department of Anesthesiology and Reanimation, Health Science University Sultangazi Haseki Training and Research Hospital, Istanbul, Türkiye; 3 Department of Anesthesiology and Reanimation, Kolan International Hospital, Istanbul, Türkiye; 4 Department of Forensic Medicine, Ankara Medipol University, Ankara, Türkiye; 5 Department of Anesthesiology and Reanimation, Istanbul Yeni Yüzyıl University Medicine Faculty, Istanbul, Türkiye

**Keywords:** end-of-life care, ethics, intensive care physicians, palliative care, physician preferences

## Abstract

**Introduction:**

Intensive care physicians are uniquely positioned to understand the benefits and burdens of life-prolonging treatments. This study aimed to examine the personal end-of-life preferences of intensive care physicians when facing a hypothetical incurable terminal illness and to evaluate the role of palliative care within these preferences.

**Materials and methods:**

In this descriptive cross-sectional study, a 17-item survey instrument (12 close-ended and 5 open-ended questions) was administered electronically to anesthesiology and reanimation specialists working in public and private hospitals in Istanbul. Quantitative data were analyzed using descriptive statistics, while qualitative responses were evaluated through thematic analysis. Content validity of the questionnaire was assessed using established content validity index methodology.

**Results:**

A total of 296 physicians participated (response rate: 28.8%). The mean age was 43.79 ± 7.54 years, and the mean duration of specialist practice was 12.34 ± 9.44 years. Refusal rates for aggressive life-prolonging interventions exceeded 70% across most modalities, with the highest refusal observed for cardiopulmonary resuscitation (88.5%). Only 0.7% of respondents indicated a preference to spend the end of life in a palliative care unit. Among physicians who had previously made end-of-life decisions for first-degree relatives, only 1.3% reported utilization of palliative care services.

**Discussion:**

Intensive care physicians strongly favor comfort-focused and palliative-oriented approaches for themselves at the end of life. However, the extremely low preference for palliative care units highlights a substantial gap between personal values and the current healthcare system. Strengthening palliative care integration, education, and policy frameworks is urgently needed.

## 1 Introduction

End-of-life care represents one of the most ethically, clinically, and organizationally complex domains of modern healthcare. The global increase in life expectancy and the growing burden of chronic and life-limiting diseases have markedly intensified the demand for palliative care services worldwide (1, 2). Despite this growing need, palliative care services remain insufficiently integrated into healthcare systems in many countries, including Türkiye, where limited capacity, regulatory ambiguities, and low public awareness continue to hinder effective implementation (3, 4).

Intensive care units (ICUs) frequently become the default care setting for patients in the terminal phase of illness (5). However, a substantial body of evidence indicates that aggressive treatments administered near the end of life provide limited benefit in terms of survival and often compromise quality of life (6–9). Consequently, the personal end-of-life preferences of intensive care physicians who routinely witness the outcomes of such interventions offer a uniquely informed perspective and an important reference point for ethical and clinical discussions.

Previous international studies have consistently shown that physicians tend to avoid aggressive life-sustaining treatments for themselves and prefer palliative-oriented approaches emphasizing comfort and dignity (10–13). Data from Türkiye on this subject remain limited. This study aimed to explore the end-of-life preferences of intensive care physicians working in Istanbul, where palliative care services are relatively more developed compared to other regions, and to assess the perceived role of palliative care within these preferences.

Global variations in end-of-life decision-making within intensive care units are heavily influenced by country-specific cultural frameworks, religious traditions, and legal structures. While Northern and Western European nations have established highly standardized, legally binding statutory frameworks for treatment limitations, Mediterranean and Middle Eastern societies tend to rely more heavily on family-centered paternalism. This cultural and legal variation underscores the necessity of evaluating these dynamics in metropolitan Turkish contexts, where healthcare resources and cultural shifts coexist.

## 2 Materials and methods

### 2.1 Study design and ethical approval

This descriptive cross-sectional study was approved by the Istanbul Yeni Yüzyıl University Ethics Committee (Approval date: 01.07.2019; Decision No: 07/19). The study was conducted in accordance with the principles of the Declaration of Helsinki.

### 2.2 Participants and questionnaire

We adapted an electronic 17-item survey instrument from validated international surveys, specifically drawing from the ETHICATT and PROPEL studies, and tailored it to the Turkish clinical and legal framework. The items span four primary domains: demographics, personal clinical preferences under terminal conditions, organ donation, and attitudes toward death. To prevent propositional response bias, we drafted the questions in Turkish using neutral, standard interrogative phrasing.

To establish content validity, a panel of 8 independent anesthesiology and reanimation specialists from public, private, and university clinics evaluated the relevance of each item on a 4-point scale (1 = not appropriate, 4 = highly appropriate). The Item-Level Content Validity Index (I-CVI) for all 17 items ranged from 0.88 to 1.00, exceeding the acceptable threshold of 0.78 for an 8-expert panel (14). Scale-level content validity was calculated using the averaging and universal agreement methods, yielding S-CVI/Ave = 0.94 and S-CVI/UA = 0.86, respectively, indicating excellent expert consensus. Prior to full deployment, a pilot study was conducted with a convenience sample of 10 anesthesiology residents (excluded from final analysis) to confirm comprehensibility and linguistic clarity; the average completion time was determined to be 6 to 8 min. To address missing data, pairwise deletion was utilized during statistical analysis, ensuring that missing responses to individual items did not exclude entire participant records.

All personal clinical preferences were evaluated using a standardized, verbatim hypothetical scenario: “Eğer kür şansı olmayan, yaşam beklentisi sınırlı ve iyileşmesi mümkün olmayan terminal dönemde bir hastalığa yakalansaydınız.” (“If you were diagnosed with a terminal, incurable illness with a limited life expectancy and no chance of cure.”)

Regarding internal consistency testing, calculating a global Cronbach’s alpha (*α*) for this instrument is psychometrically inappropriate. Cronbach’s alpha assumes a single, continuous, latent construct under the assumption of tau-equivalence. Because this 17-item survey instrument comprises independent, nominal, and categorical items addressing highly heterogeneous domains—ranging from demographic metrics and preferred routes of artificial nutrition to legal organ donor status and qualitative concerns about dying—there is no underlying unidimensional construct. Forcing an alpha calculation on such a diverse, multi-domain tool would yield a mathematically invalid and misleading coefficient. Instead, instrument stability and validation are supported by the quantitative content validity indices and pilot testing.

### 2.3 Data collection and analysis

Survey responses were collected anonymously at the end of the designated response period. Descriptive statistics, including frequencies, percentages, means, and standard deviations (SD), were calculated for all variables. Associations between demographic or occupational factors and end-of-life preferences were examined using chi-square (χ^2^) tests or Fisher’s exact tests, as appropriate. Multivariable logistic regression models were then constructed to identify independent predictors, and adjusted odds ratios (aOR) with 95% confidence intervals (CIs) were reported. Statistical significance was defined *a priori* as *p* < 0.05.

Qualitative text from the 5 open-ended questions was analyzed using Braun and Clarke’s reflexive thematic analysis framework (15). Illustrative quotes represent verbatim English translations of Turkish responses, subjected to standard back-translation validation and light grammatical editing to preserve semantic and emotional integrity. Two researchers independently read and coded the transcripts, utilizing a collaborative, interpretative coding approach. Inter-coder agreement was assessed, yielding a strong Cohen’s kappa coefficient (ĸ = 0.84) with coding discrepancies resolved through consensus. Conceptual thematic saturation was achieved systematically after the analysis of 220 open-ended responses, at which point no new conceptual codes or themes were identified.

## 3 Results

### 3.1 Personal end-of-life treatment preferences

Of the 1,029 physicians invited, 296 completed the survey, yielding a response rate of 28.8%. Participants had a mean age of 43.79 ± 7.54 years (range: 27–72) and a mean specialist experience of 12.34 ± 9.44 years (range: 1–40), indicating a predominantly mid-career cohort.

When presented with a hypothetical scenario involving an incurable terminal illness, respondents demonstrated a strong tendency to refuse aggressive interventions as presented in Table 1. High-risk major surgical procedures with minimal expected benefit were declined by 80.1% of participants. Similarly, 83.1% rejected non-curative palliative chemotherapy. Long-term mechanical ventilation, including intubation and tracheostomy, was refused by 75.7%, while 73.3% declined extracorporeal life-support modalities such as dialysis or ECMO. Cardiopulmonary resuscitation showed the highest refusal rate, at 88.5%.

**Table 1:** Survey questions and participants’ responses.

Domain	Item / Question	Response Categories	n (%) or Mean ± SD (Min–Max)
Demographics	Age (years)	—	43.79 ± 7.54 (27–72)
	Years as a specialist	—	12.34 ± 9.44 (1–40)
Personal Clinical Preferences*	Acceptance of high-risk major surgery with very limited potential benefit	Yes / No	59 (19.9) / 237 (80.1)
	Acceptance of palliative chemotherapy if clearly non-curative	Yes / No	50 (16.9) / 246 (83.1)
	Acceptance of intubation, tracheostomy, and long-term mechanical ventilation in irreversible respiratory failure	Yes / No	72 (24.3) / 224 (75.7)
	Request for extracorporeal life-support (dialysis, hemofiltration, ECMO) in vital organ failure	Yes / No	79 (26.7) / 217 (73.3)
	Desire for CPR under these conditions	Yes / No	34 (11.5) / 262 (88.5)
Preferences for Relatives	Would you make the same decisions for your spouse/children/parents?	Yes / No / Not sure	136 (45.95) / 21 (7.1) / 139 (46.95)
Organ donation	Registered organ donor	Yes / No	141 (47.6) / 155 (52.4)
	Opinion on organ donation for first-degree relatives	In favor / Against	245 (82.7) / 51 (17.3)
Preferred Nutrition Route	Preferred nutrition if oral intake impossible	Nasogastric tube (NG)	32 (10.8)
		Intravenous nutrition (IV)	69 (23.3)
		Percutaneous endoscopic gastrostomy (PEG)	173 (58.5)
		Other methods	22 (7.4)
Preferred Place of Death	Preferred place for final days of life	At home with family	237 (80.1)
		By the seaside with loved ones	20 (6.8)
		In nature, calm and quiet	15 (5.1)
		Alone	8 (2.4)
		Hospital (not ICU)	7 (2.4)
		While traveling	4 (1.01)
		No preference	2 (0.7)
		Palliative care unit^†^	2 (0.7)
		Euthanasia	1 (0.34)
Surrogate Care Decisions	Ever made an end-of-life decision for a relative	Yes / No	83 (28.0) / 213 (72.0)
	If yes: clinical care pathway chosen	Symptomatic care at home	36 (43.4)
		Mandatory ICU, no life support, no CPR	20 (24.1)
		Mandatory ICU with life support, later regretted	8 (9.6)
		Hospital care, refused ICU/CPR	8 (9.6)
		Full support including CPR until death	6 (7.2)
		Asked patient and followed their decision	4 (4.8)
		Palliative care center	1 (1.3)
Attitudes toward death	Fearful/anxious about death	Yes / No	156 (52.7) / 112 (37.8) [Other/No response: 28 (9.5)]
	Main source of concern about death (among those anxious)	Dying in ICU while conscious	64 (41.0)
		Pain, dyspnea, suffering	38 (24.4)
		Leaving loved ones/children behind	22 (14.1)
		Dying young / sadness for young deaths	13 (8.3)
		Uncertainty after death	12 (7.7)
		Not receiving appropriate treatment	5 (3.2)
		Not receiving full support until the end	1 (0.6)
		Not dying in a palliative care setting	1 (0.6)
		Not dying in a palliative care setting	1 (0.6)

*Assessed under the assumption of an incurable disease with limited life expectancy. † Only one participant explicitly requested palliative care by name. Multiple responses allowed.

Regarding organ donation, 47.6% of participants reported being registered organ donors under national law, indicating their legal willingness to donate their organs upon death. Preferences regarding end-of-life nutrition varied, with percutaneous endoscopic gastrostomy being the most frequently preferred option (58.5%), followed by intravenous nutrition (23.3%) and nasogastric feeding (10.8%).

A pronounced preference for dying at home with family members was observed (80.1%). Only 2.4% preferred to remain in hospital, and merely 0.7% expressed a preference for a palliative care unit.

Among the 83 physicians (28%) who had previously made end-of-life decisions for relatives, though the survey did not differentiate between surrogate decisions for incapacitated relatives and collaborative recommendations for relatives retaining cognitive capacity, home-based symptomatic care was the most commonly reported choice (43.4%). Only one physician (1.3%) reported referral to a palliative care center.

### 3.2 Bivariate and multivariable predictors of preferences

Bivariate analyses revealed that professional experience significantly influenced personal end-of-life choices. Physicians with ≥10 years of specialist experience were significantly more likely to refuse CPR (χ^2^ = 7.42, *p* = 0.006) and reject long-term mechanical ventilation (χ^2^ = 9.15, *p* = 0.002) than less experienced colleagues.

In multivariable logistic regression models, having ≥10 years of specialist experience remained a strong independent predictor of CPR refusal (αOR = 2.84, 95% CI: 1.34–6.02, *p* = 0.007). Prior personal experience making end-of-life decisions for a relative was also a strong independent predictor of personal refusals, including the rejection of aggressive mechanical ventilation (αOR = 2.96, 95% CI: 1.41–6.22, *p* = 0.004) and high-risk major surgery (αOR = 2.44, 95% CI: 1.18–5.06, *p* = 0.016). Conversely, physicians reporting subjective anxiety or fear of death were significantly less likely to refuse CPR (αOR = 0.42, 95% CI: 0.21–0.85, *p* = 0.016). These predictors are detailed in Table 2.

**Table 2:** Bivariate and multivariable associations predicting clinical and personal end-of-life preferences (*n* = 296).

Predictor variable	Clinical preference dependent variable	Statistical test	Coefficient / Metric	*p* value
Gender (Female vs. Male)	Approval of formal clinical DNR order legalization	Chi-Square	*χ*2 = 7.11	*p* < 0.01
Professional Experience (≥10 vs. <10 years)	Opinion that CPR should not be universally performed	Chi-Square	*χ*2 = 9.85	*p* < 0.01
Professional Experience (≥10 vs. <10 years)	Personal refusal of CPR under terminal conditions	Logistic Regression	*aOR* = 2.84 (95% CI: 1.34–6.02)	*p* = 0.007
Clinical Exposure (>50% vs. <10% terminal ICU patients)	Personal preference for DNR/DNI limit-of-care orders	Fisher’s Exact	Odds Ratio: 2.15	*p* < 0.05
Prior Family Decision (Yes vs. No history)	Personal refusal of aggressive mechanical ventilation	Logistic Regression	*aOR* = 2.96 (95% CI: 1.41–6.22)	*p* = 0.004
Prior Family Decision (Yes vs. No history)	Personal refusal of high-risk major surgery	Logistic Regression	*aOR* = 2.44 (95% CI: 1.18–5.06)	*p* = 0.016
Subjective Belief System (Atheism/Non-religious vs. Muslim)	Approval of DNR based strictly on autonomous patient request	Multivariable Logistic	*aOR* = 3.10 (95% CI: 1.25–7.68)	*p* < 0.05
Subjective Anxiety (Anxious/Fearful vs. Natural/Acceptance)	Personal refusal of CPR under terminal conditions	Logistic Regression	*aOR* = 0.42 (95% CI: 0.21–0.85)	*p* = 0.016

To ensure methodological transparency regarding the multivariable regression analyses, we clarify here the origin of the five key variables analyzed that were not explicitly listed as numbered, close-ended items in the supplementary print-ready preferences document: 1. Gender: Gathered automatically as a mandatory demographic screening variable on the digital landing page of the electronic survey login screen. 2. Clinical Exposure: Gathered during the professional profiling screen on the login page by asking: “What proportion of the patients admitted to your primary ICU are terminally ill? [] < 10% [] 10–50% [] > 50%.” 3. Religious Belief System, Approval of DNR Legalization, and Approval of Autonomous DNR Requests: These three sensitive and legally complex items were deliberately excluded from the quantitative checkbox questionnaire to avoid defensive non-response or social desirability bias in the restrictive Turkish legal landscape. Instead, they were captured qualitatively through thematic coding of the expansive open-ended comments in Question 17, and subsequently converted into nominal binary/categorical variables for the multivariable models. For complete replication, these questions have been formalized as supplemental items: Personal Belief System: “Which of the following best describes your worldview or belief system? [] Muslim [] Atheist / Non-religious [] Other [] Prefer not to answer.” DNR Legalization: “Do you support the formal legal codification and statutory recognition of Do-Not-Resuscitate (DNR) orders in Türkiye? [] Yes [] No [] Undecided.” Autonomous Requests: “Do you believe a competent patient’s autonomous request for a DNR order should be legally binding, regardless of clinical prognosis? [] Yes [] No [] Undecided”.

### 3.3 Qualitative depth and reflexive thematic framework

Reflexive thematic analysis of the five open-ended items yielded three core, consolidated themes capturing the clinical, psychological, and systemic experiences of end-of-life care in the ICU. These themes, along with subthemes, inter-coder agreement, coding saturation indices, and illustrative verbatim quotes, are presented in Table 3.

**Table 3:** Qualitative reflexive thematic analysis framework (*n* = 296).

Core consolidated theme	Primary associated subthemes	Illustrative verbatim quotes (Translated from Turkish)	Coding saturation status (*n* = 220)
Theme 1: Dread of the monitored death trajectory (The “ICU Syndrome”)	Depersonalization of high-noise, high-intensity clinical spaces. Prolongation of physiological dying over cognitive recovery. Instrumentalization of the human body near death.	“I have spent my entire professional life maintaining failing organs on machines. I do not want my own final days to be defined by the relentless beeping of monitors, physical restraint, and the sterile coldness of an ICU.” “We often confuse prolonging life with extending active dying. I demand that machines be left off when cognitive recovery is impossible.”	Conceptual thematic saturation systematically achieved after analysis of 220 responses.
Theme 2: Sociocultural duty and filial guilt	Societal stigma of institutionalized elder care. Religious mandates of preserving life at all costs. Paternalistic family decision structures bypassing autonomy.	“When my father was terminally ill, my medical training screamed that further interventions were futile. Yet, the pressure from family and neighbors to ‘fight’ created an unbearable conflict.” “As physicians, we know the statistics, but as Turkish children, letting go is equivalent to abandonment.”	Conceptual thematic saturation systematically achieved after analysis of 220 responses.
Theme 3: Clinical defensiveness and legal vacuum	Criminal liability under active penal statutes. Inability to legally enforce documented wishes. Lack of standardized institutional decision structures.	“We conceptually support palliative principles, but our healthcare system forces a binary choice: either you die in an over-utilized ICU or you go home to die with zero support.” “Without clear legal protections for DNR orders, we are forced to practice defensive medicine, resuscitating patients we know cannot benefit.”	Conceptual thematic saturation systematically achieved after analysis of 220 responses.

## 4 Discussion

This study examined the end-of-life preferences of intensive care physicians working in Istanbul and demonstrated that the vast majority tend to avoid aggressive and life-prolonging interventions for themselves. Our findings suggest that frequent professional exposure to death and dying processes leads physicians to frequently develop a cautious, comfort-oriented, and realistic approach to their own end-of-life care. This tendency is consistent with the general patterns reported in international literature. For instance, the ETHICATT study showed that intensive care physicians across Europe preferred to die outside the ICU whenever possible, favoring environments such as home or hospice settings with lower levels of medical intervention (16). Our results confirm that physicians in Istanbul-Türkiye display similar inclinations.

The relationship between personal preferences and clinical practice has become an increasingly explored topic in end-of-life care research. The PROPEL study demonstrated that many physicians project their personal end-of-life preferences onto patient care and that a substantial proportion of clinical recommendations are influenced by these subjective values (11). This phenomenon aligns with the concept known in health ethics literature as the “ethical mirror.” The ethical mirror refers to a reflective mechanism through which physicians consciously reassess their own values, fears of death, professional identities, and personal life experiences, thereby integrating these reflections more consistently into clinical decision-making (17). This reflective process highlights that end-of-life decisions are shaped not only by clinical parameters but also by physicians’ ethical positioning and personal perspectives.

However, the PROPEL study also reported an important discrepancy: physicians often consider options such as palliative sedation or euthanasia more acceptable for themselves, while approaching these same interventions with greater caution when caring for patients (11). This inconsistency reflects the delicate balance physicians attempt to maintain between respect for patient autonomy, the principle of non-maleficence, and their professional responsibilities.

Notably, our study identified a significant discrepancy between what intensive care physicians desire for themselves and what they are willing to decide for their first-degree relatives. While the vast majority (88.5%) refused CPR for themselves, only 45.9% answered ‘Yes’ when asked if they would make the same comfort-oriented choices for their spouse, children, or parents, with 47.0% reporting being ‘Not sure’. This hesitation reveals a critical ethical tension. In a deeply collectivist and family-oriented society like Türkiye, surrogate decision-makers are heavily influenced by traditional expectations, Islamic filial duties, and profound fear of social stigma, making them highly reluctant to limit aggressive, life-prolonging treatments for loved ones (18, 19). Culturally, the care of elderly or terminally ill parents is regarded as an absolute moral and religious duty (filial responsibility/evlatlık vazifesi) rather than a voluntary option, where families fear social condemnation and profound guilt if they do not insist on “maximum care” until the very end (18, 20). In this collectivist structure, medical decision-making is relational and family-centered, often directed by patriarchal family hierarchies that override individual patient autonomy in favor of clinical or familial paternalism (21, 22). When critical care specialists act as surrogate decision-makers for their own relatives, they are caught between their clinical understanding of medical futility and these powerful sociocultural expectations (23, 24). This conflict explains why physicians default to highly interventionist choices for their loved ones, even when they would vigorously refuse those same interventions for themselves.

International research has consistently demonstrated significant cross-national differences in end-of-life practices within intensive care units. The Ethicus and Ethicus-2 studies revealed that variations among countries are strongly influenced by cultural norms, religious structures, the institutional use of ethical guidelines, and healthcare system organization (25, 26). For example, physicians in the United States tend to rely more frequently on formal ethical guidelines, whereas decision-making processes in many European countries are more collective and interdisciplinary (25, 27). A national survey conducted in Poland found that the majority of ICU physicians supported withholding and withdrawing life-sustaining treatments, yet firmly rejected active life-ending interventions (28). These findings underscore that end-of-life decisions are shaped not solely by medical knowledge, but also by cultural frameworks and ethical codes embedded within healthcare systems.

In the post-COVID-19 era, end-of-life care in intensive care units has become a prominent global discussion topic. Guidelines issued by the European Society of Intensive Care Medicine (ESICM) emphasize the systematic integration of palliative care into ICU practice, early palliative care assessment, structured communication with patients and families, and the strengthening of ethical support mechanisms (5, 29). Despite these recommendations, surveys conducted across Europe between 2023 and 2024 indicate that intensive care professionals continue to face communication challenges, limited access to ethical consultation, uncertainty regarding palliative care integration, and resource constraints (30). OECD reports further highlight that palliative care capacity—particularly in societies with a strong preference for home death—remains insufficient to meet demand, even in high-income countries (30–34). These data demonstrate that the mismatch between end-of-life preferences and healthcare infrastructure is not confined to developing countries but is also evident in well-resourced health systems.

Within the Turkish context, our findings both align with and diverge from international literature in important ways. While most participating physicians reported a desire to avoid dying in the ICU and to refrain from aggressive treatments, only 0.7% expressed a preference to die in a palliative care unit. This proportion is markedly lower than figures reported in European countries where hospice use and home-based end-of-life care are more prevalent (16, 25). In Western and Northern European countries (e.g., the United Kingdom, Belgium, and the Netherlands), approximately 40 to 60% of terminally ill patients access home-care or specialized hospices, and a high proportion of physicians regularly utilize formal palliative pathways. This stands in stark contrast to our cohort, where only 0.7% preferred a palliative care unit for themselves and a mere 1.3% of those who made decisions for relatives utilized palliative care services. This striking discrepancy reflects both infrastructural deficiencies and cultural values.

Furthermore, 15.3% of our cohort rejected traditional environments (home or hospital) entirely, preferring instead to spend their final days by the seaside (6.8%), in nature (5.1%), alone (2.4%), or while traveling (1.01%). These specific environmental categories derived during our pilot phase from local cultural assumptions represent a psychological ‘counter-response’ to the highly medicalized, sterile, and high-noise ICU environment in which these physicians work daily, highlighting a deep desire to escape clinical spaces.

This striking discrepancy suggests significant deficiencies in the visibility, accessibility, and systemic integration of palliative care services in Türkiye. Indeed, national studies indicate that palliative care services in Türkiye remain in a developmental phase, with limited inter-institutional coordination and inconsistent implementation (3, 4).

Notably, although physicians in our study demonstrated conceptual openness toward palliative approaches, they reported limited application of these principles in clinical practice. This gap highlights an urgent need for systemic and structural reforms. Key areas requiring sustained improvement include palliative care education, referral pathways, legal clarity regarding end-of-life decisions, and the availability of ethics consultation services. International evidence suggests that structured palliative care education programs enhance physicians’ confidence, ethical consistency, and decision-making capacity—both in personal end-of-life preferences and in patient care (5, 35). Given the emotional burden and moral distress associated with end-of-life decision-making, such educational initiatives are also crucial for reducing physician burnout and moral injury.

The legal environment in Türkiye creates direct, moral-legal conflicts for intensive care physicians. While Türkiye is a signatory to the Oviedo Convention (which binds national law to respect a competent patient’s right to refuse treatment), standalone statutory frameworks for living wills or advance directives do not exist. The Patient Rights Regulation (Article 25) technically guarantees the right to refuse or discontinue treatment (36). However, this is heavily restricted by Article 13 of the same regulation, which explicitly prohibits euthanasia, stating that nothing can be done or requested that is life-threatening or causes death (36).

Consequently, Do-Not-Resuscitate (DNR) and Do-Not-Intubate (DNI) orders are unrecognized and have no legal validity in Türkiye. Passive euthanasia (withholding or withdrawing life-support) is legally hazardous. The Turkish Penal Code (TPC) Article 81 prosecutes active life-ending actions as deliberate murder (punishable by 24–30 years in prison), while Article 83 classifies treatment withdrawal or limitation as homicide by omission (36). Despite these severe criminal risks, national literature shows that between 40.0 and 65.9% of Turkish physicians have restricted futile treatments in the ICU. To navigate this high-risk legal vacuum, physicians rely on unstructured, informal clinical consensus and verbal agreements among colleagues, shielding themselves from litigation while quietly prioritizing patient comfort.

## 5 Limitations

There are several limitations to this study. First, while current personal preferences are not subject to recall bias, retrospective evaluations regarding past end-of-life decisions made for first-degree relatives (Item 13) are susceptible to recall and cognitive rationalization bias, as physicians may reconstruct memories to reduce emotional distress. Second, the response rate of 28.8%, while typical for written surveys of medical specialists, introduces a risk of non-response and self-selection bias. Physicians with a pre-existing interest in bioethics or those who have experienced severe moral distress are likely overrepresented, whereas clinicians who default to defensive, highly interventionist practices may have systematically opted out. This could potentially skew our results toward a higher reported refusal of life-sustaining treatments.

Third, the study population was limited to anesthesiology and reanimation specialists working in Istanbul. Istanbul is the most economically and socioeconomically developed metropolis in Türkiye, possessing the highest concentration of tertiary medical centers and advanced palliative care infrastructure; thus, findings cannot be generalized to rural or semi-urban provinces where different resource limitations and more conservative cultural frameworks exist. Finally, we identified no formal physician-led end-of-life or ‘right-to-die’ advocacy groups in Türkiye that could have systematically influenced the cohort’s opinions. Despite these limitations, this study remains a rare and valuable investigation of critical care specialists’ end-of-life preferences.

## 6 Conclusion

Intensive care physicians in Istanbul, Türkiye, predominantly favor comfort-focused, non-invasive end-of-life care for themselves, with a striking 80.1% preferring home-based dying. However, their preference for and actual utilization of institutionalized palliative care units remains extremely low (0.7%). Rather than indicating a direct ‘systemic mismatch’ with healthcare structures, this pattern reflects a strong cultural desire to spend final days in a familiar, non-institutionalized home environment, alongside a potential lack of familiarity with or trust in the current institutional palliative care infrastructure. Expanding and funding home-based palliative care under national social security, establishing clinical ethics guidelines, and directly researching physicians’ perceptions of institutionalized palliative care are vital to aligning end-of-life clinical practices with physician and patient preferences.

## Data Availability

The original contributions presented in the study are included in the article/Supplementary material, further inquiries can be directed to the corresponding author.
