# Supplementary material for: How do intensive care physicians wish to die? A cross-sectional study on end-of-life preferences and the role of palliative care
Source: Front Med (Lausanne). 2026 Jul 6;13:1872490. doi: 10.3389/fmed.2026.1872490 (PMC13381785; doi:10.3389/fmed.2026.1872490)
Supplement: Supplementary file 1 [file Data_Sheet_1.PDF]

Questionnaire: Physicians' End-of-Life Treatment Preferences and Attitudes Toward Death

Section 1. Demographic and Professional Information

1. Age:
Please write your age in years:
_____ years

2. Years as a specialist:
How many years have you been working as a specialist physician?
_____ years

Section 2. Personal End-of-Life Treatment Preferences

Please answer the following questions by considering a situation in which you have a **terminal or irreversible medical condition**, with very limited chance of recovery or meaningful clinical benefit. Please select one option unless otherwise stated.

3. Would you accept high-risk major surgery if the expected medical benefit were very limited?

☐ Yes

☐ No

4. Would you accept palliative chemotherapy if it were clearly non-curative?

☐ Yes

☐ No

5. In the case of irreversible respiratory failure, would you accept intubation, tracheostomy, and long-term mechanical ventilation?

☐ Yes

☐ No

6. In the case of vital organ failure, would you request extracorporeal life-support methods such as dialysis, hemofiltration, or ECMO?

☐ Yes

☐ No

Section 3. Preferences for First-Degree Relatives

7. Would you make the same end-of-life treatment decisions for your first-degree relatives, such as your spouse, children, or parents, as you would for yourself?

☐ Yes

☐ No

☐ Not sure

Section 4. Organ Donation

8. Are you a registered organ donor?

☐ Yes

☐ No

9. What is your opinion about organ donation for your first-degree relatives?

☐ In favor

☐ Against

Section 5. Preferred Nutrition Method if Oral Intake Is Impossible

10. If oral intake became impossible in an end-of-life situation, which nutrition method would you prefer?

Please select one option.

☐ Nasogastric tube

☐ Intravenous nutrition

☐ Percutaneous endoscopic gastrostomy

☐ Other: Please specify: ______________________

Section 6. Cardiopulmonary Resuscitation

11. Under the conditions described above, would you want cardiopulmonary resuscitation to be performed in the event of cardiac arrest?

☐ Yes

☐ No

Section 7. Preferred Place for Final Days of Life

12. Where would you prefer to spend your final days of life?

Please select the option that best reflects your preference.

☐ At home with family

☐ By the seaside with loved ones

☐ In nature, in a calm and quiet environment

☐ Alone

☐ In hospital, but not in the intensive care unit

☐ While traveling

☐ No preference

☐ In a palliative care unit

☐ Euthanasia

☐ Other: Please specify: ______________________

Section 8. Experience in End-of-Life Decision-Making for a Relative

13. Have you ever made an end-of-life care or treatment decision for a relative?

☐ Yes

☐ No

If your answer is "Yes," please answer Question 14.

14. Which of the following best describes the decision or care approach?
Please select one option.

☐ Symptomatic care at home
☐ Mandatory ICU admission, but without life support and without CPR
☐ Mandatory ICU admission with life support, later regretted
☐ Hospital care, but ICU admission and CPR were refused
☐ Full support, including CPR, until death
☐ The patient was asked, and their decision was followed
☐ Palliative care center, including analgesia and nutrition
☐ Other: Please specify: ______________________

Section 9. Attitudes Toward Death

15. Which of the following best describes your attitude toward death?
Please select one option.

☐ I feel fearful or anxious about death
☐ I accept death as natural and inevitable, and I am not afraid
☐ Other: Please specify: ______________________
☐ Prefer not to answer

Section 10. Sources of Concern About Death

16. If you feel fear, anxiety, or concern about death, what is the main source of this concern?
Please select the option that best reflects your view.

☐ Dying in the intensive care unit while conscious
☐ Pain, dyspnea, or suffering
☐ Leaving loved ones or children behind
☐ Dying young, or sadness related to young deaths
☐ Uncertainty about what happens after death
☐ Not receiving appropriate treatment
☐ Not receiving full support until the end
☐ Not dying in a palliative care setting
☐ Other: Please specify: ______________________
☐ Not applicable

17. Would you like to add any comments about end-of-life care, treatment limitations, palliative care, or medical decision-making?
